# A phosphorylation switch at MRE11 links ATM-ATR and calcium signaling to safeguard stalled replication fork stability

**DOI:** 10.21203/rs.3.rs-9192680/v1

**Published:** 2026-04-22

**Authors:** Weihang (Valerie) Chai, Manobendro Ray, Chih-Chun Chang, Zhen-guo Wang, Zhiguo Li, Peter Chi

**Affiliations:** Rosalind Franklin University of Medicine and Sciences, Chicago Medical School; Rosalind Franklin University of Medicine and Sciences, Chicago Medical School; National Taiwan University; Rosalind Franklin University of Medicine and Sciences, Chicago Medical School; University of Kentucky; National Taiwan University

## Abstract

MRE11 safeguards genome stability at stalled replication forks, where its activity must be tightly controlled to prevent nascent strand DNA degradation (NSD). However, the upstream signaling mechanisms that limit NSD remain poorly defined. Here, we identify Ser649 (S649) as a previously unrecognized phosphorylation site that limits MRE11 association with stalled forks. We show that S649 phosphorylation is robustly induced by replication stress or elevated cytosolic calcium levels, and is mediated by the calcium-responsive CaMKK2-AMPKα axis in concert with ATR, but independently of CHK1. Loss of S649 phosphorylation enhances MRE11 binding to DNA and increases its association with stalled forks, driving excessive NSD, elevated DNA damage, and increased sensitivity to PARP inhibition. We find that the ATM-mediated S676/S678 phosphorylation primes S649 phosphorylation, which in turn facilitates subsequent phosphorylation of SQ/TQ sites in MRE11. Moreover, we find that CaMKK2-AMPKα activation requires ATR but is independent of ATM. Collectively, our findings reveal a hierarchical signaling mechanism that couples calcium signaling with ATM/ATR pathways to prevent NSD at stalled forks and preserve genome integrity.

## Introduction

Genome instability is a defining hallmark of cancer that drives tumor initiation, evolution, and therapeutic response ^1, 2, 3^. Replication stress, characterized by impaired replication fork progression, is a major source of genome instability. Persistent fork stalling promotes fork collapse, uncontrolled nucleolytic degradation, and double-strand break (DSB) formation, fueling chromosomal rearrangements and tumorigenesis ^4, 5, 6, 7^. To preserve genome integrity, cells use a coordinated fork protection network centered on ATR-CHK1 signaling and factors such as BRCA1/2 and Fanconi anemia proteins, which limit unwanted nucleolytic activities to stabilize stalled forks and enable restart ^8^.

One key determinant of fork stability is the MRE11 nuclease, a central component of the MRN complex (MRE11/RAD50/NBS1) that coordinates DSB repair, replication restart, and checkpoint signaling ^9, 10^. The roles of MRN in DSB repair have been extensively characterized. Within this complex, RAD50 is the ATPase-driven structural core that provides long-range DNA tethering and contributes to activation of DNA damage signaling ^11, 12^. NBS1 serves as a scaffold protein that mediates nuclear localization of the MRN complex and recruitment of DNA damage response and repair proteins like ATM, MDC1 and CtIP ^13, 14, 15, 16, 17, 18^. MRE11 is a dual-functional nuclease, exhibiting endonuclease activity on single-stranded (ss) DNA as well as processing DNA ends through its 3′ to 5′ exonuclease activity. A critical step in the repair of DSBs by homologous recombination (HR) is the generation of 3′ ssDNA overhangs, which facilitates RAD51 filament formation and homology-directed strand invasion ^19, 20, 21, 22, 23^. This early resection step requires the cooperation of CtIP, which stimulates the endonuclease activity of MRN to cleave DNA near bulky obstacles such as KU70/80 or topoisomerase II, thereby enabling resection initiation ^13, 24, 25^. After this initial resection, long-range resection is carried out by nucleases such as EXO1 and DNA2 ^26, 27^. In addition, helicases such as BLM unwind DNA and extend ssDNA tracts necessary for RAD51 loading and HR ^26, 28, 29, 30, 31^. Thus, MRN functions as an upstream regulator of HR-mediated DSB repair.

Besides its role in DSB repair, MRE11 also functions at stalled replication forks. ATM/ATR facilitates recruitment of the MRN complex to the stalled replication forks, where MRE11 processes aberrant DNA structures and promotes fork stabilization and restart ^32, 33^. While MRE11 is essential for fork processing and repair, unscheduled nuclease activity has been implicated in pathological fork degradation, especially in cells deficient in fork protection ^34, 35^. In BRCA2-deficient cells, destabilization of RAD51 filaments allows MRE11 to degrade newly synthesized DNA strands at reversed forks, resulting in replication fork instability and chromosomal aberrations ^35 36, 37^. Degradation of nascent DNA by MRE11 contributes to genomic instability and synthetic lethality with PARP inhibition ^34, 38^. Despite these insights, the mechanisms that restrain MRE11 nuclease activity at stalled replication forks remain poorly understood.

Post-translational modifications (PTMs), particularly phosphorylation, are critical in regulating DNA repair proteins in response to replication stress ^39, 40, 41^. Several studies have shown that phosphorylation of MRE11 modulates the MRN complex activity by influencing DNA binding, nuclease activity, and chromatin association. In particular, ATM phosphorylation of MRE11 promotes dissociation of MRN from chromatin, thereby restricting excessive DNA end processing ^42^. During HR, ATM-dependent phosphorylation of MRE11 at S676/S678 fine-tunes DNA end processing by restricting EXO1 from pathological over-resection ^43^. Consistent with this model, ATM phosphorylation of MRE11 at S676/S678 promotes dissociation of inhibitory C1QBP from MRE11/RAD50/C1QBP complex, enabling formation of the MRN complex and its recruitment to DSBs ^44^. In addition, ribosomal S6 kinase (RSK) can phosphorylate MRE11 predominantly at S676, reducing MRE11 DNA binding and attenuating ATM signaling ^45^. In addition to ATM/ATR signaling, phosphorylation of MRE11 on non-SQ/TQ sites regulates MRN activity. Specifically, during G2/M phase transition, PLK1 (Polo-like kinase 1) phosphorylates MRE11 at S649, priming CK2-dependent phosphorylation at S688 that inhibits MRN loading onto damaged DNA to promote checkpoint recovery and ensure mitotic entry ^46^.

At stalled forks, recent work has revealed that MRE11 is subject to sequential phosphorylation by ATM and ATR, which regulates DNA binding and promotes MRE11 dissociation from stalled forks to prevent extensive degradation of nascent DNA by MRE11 ^47^. This study established a model where the MRN complex, upon being recruited to the single-ended double-strand breaks (se-DSBs), activates ATM, which in turn phosphorylates MRE11 at the S676/S678 motif ^47^. This ATM-mediated phosphorylation triggers subsequent ATR-dependent phosphorylation of SQ/TQ motifs of MRE11, causing MRE11 dissociation from reversed forks. Thus, ATM- and ATR-mediated phosphorylation of MRE11 controls the extent of fork resection and protects fork stability. However, the upstream regulatory events and the potential role of non-SQ/TQ phosphorylation sites on MRE11 in modulating MRE11 activity remain largely unknown.

Aside from the well-established ATR-CHK1 checkpoint signaling, recent studies have uncovered the calcium (Ca^2+^)-responsive CaMKK2-AMPKα signaling axis in safeguarding stressed replication forks. When replication is perturbed, damaged DNA fragments are released from the nucleus into the cytoplasm. This cytosolic DNA activates the cGAS-STING pathway, which triggers TRPV2 channel-mediated Ca^2+^ release from the endoplasmic reticulum (ER) ^48, 49^. Afterwards, elevated cytosolic Ca^2+^ levels activate CaMKK2 (calcium/calmodulin dependent protein kinase kinase 2), which in turn activates AMPKα, leading to phosphorylation of EXO1. This PTM prevents aberrant resection of nascent DNA strands by EXO1, thus ensuring fork stability ^48, 49, 50^. Besides EXO1, CaMKK2 also directly phosphorylates STN1, a key subunit of the fork protector CST (CTC1/STN1/TEN1) complex. This phosphorylation facilitates the localization of CST to stalled forks and prevents MRE11 nuclease-mediated aberrant NSD, reinforcing fork stabilization under replication stress-induced Ca^2+^ elevation ^51^.

Despite these observations, the CaMKK2-AMPKα signaling axis in genome maintenance remains largely underexplored, and the functional relationship between this signaling pathway with the canonical ATR/ATM signaling remains largely elusive. To date, only two replication fork proteins, EXO1 and STN1, are the known targets of this signaling pathway ^50, 51^. This pathway represents a non-canonical but functionally critical layer of replication stress response, and it is highly possible that additional fork proteins may be subject to phosphorylation by CaMKK2-AMPKα. In this study, we uncover that the Serine 649 residue located in the DNA-binding domain of MRE11 is phosphorylated in response to perturbed DNA replication and elevated cytoplasmic Ca^2+^ concentration. Our results show that S649 phosphorylation is abolished in CaMKK2 knockout (KO) cells and AMPKα KO cells, suggesting that phosphorylation of S649 is through a Ca^2+^-dependent CaMKK2-AMPKα pathway. *In vitro* kinase assay shows that the AMPKα kinase can directly phosphorylate the MRE11 protein. Using phospho-deficient and phospho-mimetic mutants, our *in vitro* and cell-based assays demonstrate that S649 phosphorylation impairs MRE11 DNA binding, suppresses MRE11 recruitment to stalled forks and reduces its DNA degradation activity, thereby preventing pathological NSD. Furthermore, our data show that the ATM-mediated phosphorylation at S676/S678 sites of MRE11 primes S649 phosphorylation, which in turn promotes efficient ATR-dependent phosphorylation of SQ/TQ motifs, suggesting a phosphorylation cascade that links ATM-ATR signaling with the Ca^2+^-responsive CaMKK2-AMPKα pathway. In addition, our data suggest that ATR is required for the activation of CaMKK2-AMPKα, while the CaMKK2-AMPKα pathway does not control CHK1 signaling through ATR. Our results reveal that the canonical ATM and ATR DNA damage checkpoint signaling integrates with the calcium-dependent pathway to tightly control the nucleolytic activity at stalled forks, and suggest that S649 phosphorylation serves as a molecular brake to restrict MRE11 binding to stalled forks, thereby preventing erroneous fork degradation and safeguarding genome stability.

## Results

### Replication stress induces MRE11 phosphorylation at S649

**Figure 1:**
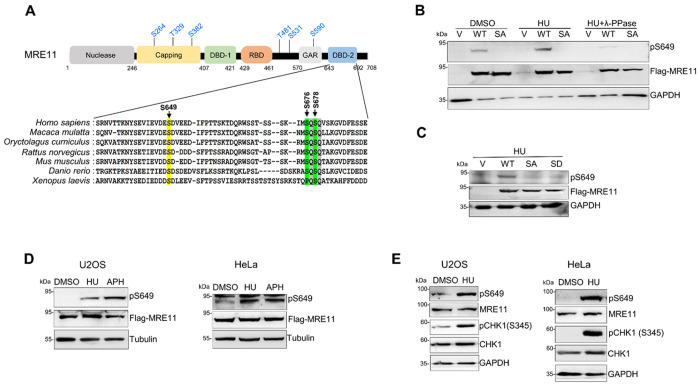
Replication stress induces phosphorylation of MRE11 at S649. **A.** Schematic representation of human MRE11 protein domains. DBD-1: DNA binding domain 1; DBD-2: DNA binding domain 2; RBD: RAD50-binding domain; GAR: Glycine-Arginine-rich domain. ATR-targeted SQ/TQ sites are indicated in blue. Sequence alignment of DBD-2 from higher eukaryotes is illustrated to show conservation of S649 (yellow) and the ATM-phosphorylated S676/S678 site (green). **B.** Western blot to detect S649 phosphorylation in response to HU treatment and the specificity of the anti-pS649 antibody. Whole-cell lysates of HEK293T cells transiently expressing FLAG-WT MRE11 or S649A mutant (SA) were analyzed following HU treatment (4 mM, 3 h) with or without λ-PPase treatment. **C.** The anti-pS649 antibody specifically recognizes WT-MRE11. Whole-cell lysates from HEK293T cells transiently expressing FLAG tagged WT-MRE11, S649A, or S649D (SD) mutants were analyzed following HU treatment (4 mM, 3 h). **D.** Western blot of pS649 in U2OS and HeLa cells transiently expressing FLAG-WT MRE11 following treatment with HU (4 mM, 3 h) or aphidicolin (2 μg/mL, 3 h). **E.** Western blot of endogenous MRE11 protein in U2OS and HeLa cells after HU treatment (4 mM, 3 h) showing that HU induces S649 phosphorylation of endogenous MRE11. All western blot images shown in this manuscript are representative blots from three independent experiments.

Previous studies have shown that MRE11 is phosphorylated at conserved residues in response to a variety of genotoxic agents (Fig. 1A) ^42, 43, 46, 47^. Sequence alignment revealed strong conservation of the S649 residue across species, suggesting functional importance (Fig. 1A). S649 resides in the second DNA binding domain of MRE11 (Fig. 1A). Since conserved serine residues in this domain play a pivotal role in regulating the stability and function of MRE11 ^42, 52^, we sought to investigate whether S649 was phosphorylated in response to replication stress. Using the phospho-specific pS649 antibody, we detected robust S649 phosphorylation in HEK293T cells expressing WT-FLAG-MRE11 upon HU treatment, suggesting that S649 phosphorylation is induced by stalled replication (Fig. 1B). λ-phosphatase treatment abolished the pS649 signal, validating that the antibody specifically recognized phosphorylation at S649 (Fig. 1B). Consistent with this, no phosphorylation signal was detected in the phospho-deficient S649A mutant, further validating the antibody specificity (Figs. 1B–C). Interestingly, the antibody did not recognize the phosphomimetic S649D mutant, possibly due to the structural alteration by the aspartate substitution that blocked antibody recognition (Fig. 1C). We then tested the replication stress-induced S649 phosphorylation in two other cell lines, human osteosarcoma cell line U2OS and cervical carcinoma cell line HeLa, to determine whether the phosphorylation is cell line specific. FLAG-MRE11 was transiently expressed in U2OS and HeLa, followed by HU or aphidicolin (APH) treatment. Robust S649 phosphorylation signal was observed after HU or APH treatment (Fig. 1D). Thus, the S649 phosphorylation induced by replication stress is not cell line-specific. In addition, we detected S649 phosphorylation of endogenous MRE11 protein in U2OS and HeLa cells upon HU treatment (Fig. 1E). Taken together, these results indicate that MRE11 is phosphorylated at S649 in response to replication stress.

### S649 phosphorylation inhibits NSD under replication stress

**Figure 2:**
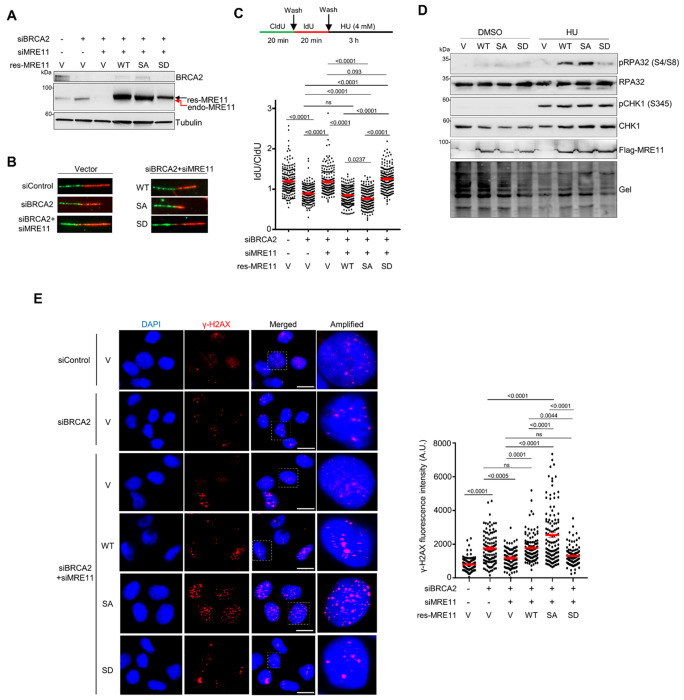
S649 phosphorylation protects NSD under replication stress. **A.** Western blot showing BRCA2 and MRE11 co-depletion and the expression of siRNA-resistant FLAG-WT-MRE11, FLAG-S649A, and FLAG-S649D mutants in U2OS cells. Endogenous MRE11 is indicated by a red arrow, and exogenously expressed FLAG-MRE11 is indicated by a black arrow. **B.** DNA fiber analysis for measuring NSD in U2OS cells stably expressing RNAi-resistant FLAG-tagged WT-MRE11, S649A, and S649D with concurrent knockdown of endogenous MRE11 and BRCA2. Cells were treated with HU (4 mM, 3 h). n: ~200 fibers per sample were analyzed in each experiment. Three independent experiments were performed and results from one experiment are shown. *P*: one-way ANOVA with Tukey’s post hoc test. **C.** Western blot of phosphorylated RPA32 (pS4/S8) from whole-cell lysates of U2OS cells stably expressing FLAG-WT-MRE11, S649A or S649D mutants following HU treatment (4 mM, 3 h). Total protein loading was assessed using stain-free gel imaging prior to transfer (gel). **D.** IF analysis of g-H2AX in U2OS cells stably expressing RNAi-resistant FLAG-WT-MRE11, S649A, and S649D with concurrent knockdown of endogenous MRE11 and BRCA2. Cells were treated with HU (4 mM, 3 h) and fixed with 4% paraformaldehyde for IF. Scale bars: 20 μm. Three independent experiments were performed and results from one experiment are shown. *P*: one-way ANOVA with Tukey’s post hoc test.

MRE11 is the major nuclease degrading nascent strand DNA at unprotected stalled forks ^9, 36, 37, 53^. We hypothesized that the replication stress-induced S649 phosphorylation regulated MRE11 activity at stalled forks. To test this, we generated U2OS cell lines stably expressing RNAi-resistant WT FLAG-MRE11, FLAG-S649A, or FLAG-S649D using retroviral transduction, and then depleted BRCA2 to deprotect forks. Concurrently, endogenous MRE11 was depleted with siRNA in these cells (Fig. 2A). Cells were sequentially labeled with CldU and IdU, followed by HU treatment (4 mM, 3 h), and NSD was assessed using DNA fiber analysis. As expected, BRCA2 depletion led to NSD, and co-depletion of BRCA2 and MRE11 abolished NSD, consistent with MRE11 being required for NSD (Fig. 2B). Expressing siRNA-resistant WT-MRE11 in cells co-depleted of BRCA2/MRE11 restored NSD to the level similar to singly BRCA2-depleted cells, confirming that exogenous MRE11 retained nuclease activity (Fig. 2B). Notably, S649A exhibited a slightly stronger NSD activity than WT-MRE11, whereas S649D showed complete protection in BRCA2-deficient cells (Fig. 2B). To determine whether the observed NSD resulted from end resection of fork DNA, we examined phosphorylation levels of RPA32 at S4/S8 (pS4/S8), which is a well-established marker of end resection ^54^. Upon HU treatment, both WT and S649A-expressing cells displayed robust pS4/S8 levels, while S649D-expressing cells displayed reduced pS4/S8 compared to WT- or S649A-expressing cells (Fig. 2C).

To determine whether enhanced NSD caused DNA damage, we examined DNA damage accumulation by γH2AX staining under BRCA2-depleted conditions. BRCA2 knockdown alone led to a strong increase in γH2AX intensity relative to the control knockdown, and MRE11 depletion reduced such elevated DNA damage levels (Fig. 2D). Cells re-expressing the RNAi-resistant WT-MRE11 exhibited comparable levels of DNA damage to the BRCA2-depleted condition (Fig. 2D, lane 4 vs lane 2), while S649A expression significantly exacerbated DNA damage and exhibited higher γH2AX intensity compared to WT (Fig. 2D, lane 5 vs lane 4), suggesting that loss of S649 phosphorylation leads to enhanced DNA degradation and damage at stalled forks. In contrast, re-expressing the RNAi-resistant S649D in BRCA2/MRE11 co-depleted cells reduced DNA damage levels to those observed in vector control cells (Fig. 2D, lane 6 vs lane 3). Taken together, these results suggest that S649 phosphorylation suppresses the fork degradation activity of MRE11, thereby promoting replication fork stability under replication stress.

### S649 phosphorylation regulates MRE11 association with stalled replication forks

**Figure 3:**
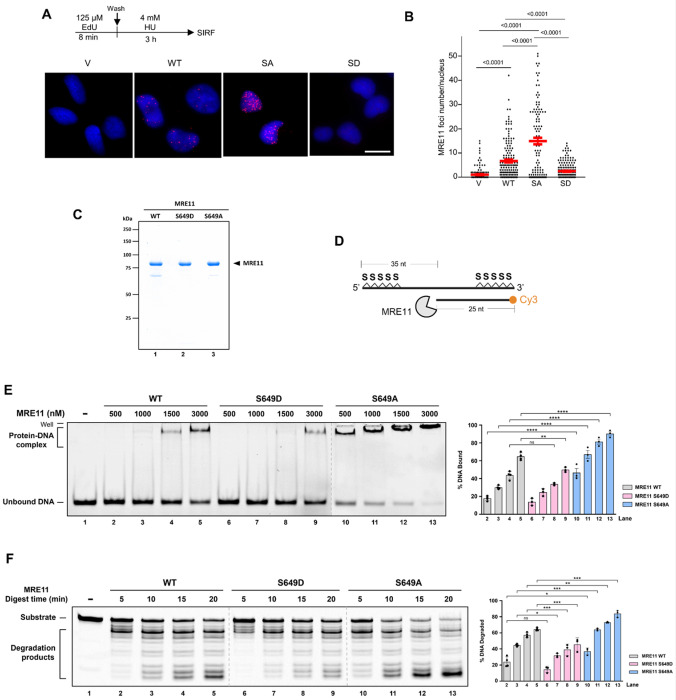
Phosphorylation at S649 controls MRE11 association with stalled forks and DNA binding and degradation *in vitro*. **A.** SIRF assay detecting FLAG-tagged WT, S649A, and S649D MRE11 at stalled replication forks in U2OS cells stably expressing the indicated constructs and treated with HU (4 mM, 3 h). Representative SIRF images are shown. Scale bars, 20 μm. **B.** Quantification of SIRF foci per cell. Foci were manually counted. n: ~150 cells per sample were analyzed in each experiment. Three independent experiments were performed and results from one experiment are shown. *P*: one-way ANOVA with Tukey’s post hoc test. **C.** Coomassie blue stained SDS-PAGE gel of purified human WT-MRE11, S649A and S649D. **D.** DNA substrate used in EMSA and MRE11-mediated DNA degradation assays. Scheme shows the nuclease activity of MRE11 in degrading 5’ Cy3-labeled substrates (25 nt + 60 nt with phosphorothioate bonds on both ends as indicated by “S”). **E.** EMSA shows enhanced DNA binding activity of S649A and reduced DNA binding activity of S649D. The 5’ Cy3-labeled substrates were incubated with the indicated concentration of MRE11 at 37°C for 30 min. Reaction products were resolved on TBE-PAGE gels. The results are graphed, and error bars represent the standard deviation (± SD) calculated from at least three independent experiments. **F.** Time-course DNA degradation assays show S649D reduces DNA degradation ability of MRE11 whereas S649A increases DNA degradation ability. The 5’ Cy3-labeled substrates were incubated with WT, S649A, or S649D protein for indicated time. Reactions were stopped by SDS and proteinase K. Samples were resolved in 27% denatured polyacrylamide gel. The results are graphed, and error bars represent the standard deviation (± SD) calculated from at least three independent experiments.

Since the S649A mutant showed strong NSD activity (Fig. 2B), we next determined whether the phosphorylation might regulate MRE11 localization to stalled forks. To investigate this, we performed the SIRF (*in situ* analysis of protein interaction at replication forks) assay ^55^ in U2OS cells stably expressing FLAG-tagged WT-MRE11, S649A or S649D after HU treatment (Fig. 3A). Interestingly, compared to WT-MRE11, S649A markedly increased its localization to stalled forks, whereas S649D displayed reduced fork localization (Fig. 3B), implying that S649 phosphorylation regulates MRE11 binding to stalled forks.

### S649 phosphorylation regulates MRE11 DNA binding and DNA degradation

To determine whether the phosphorylation directly impacted MRE11 binding to DNA, we used purified MRE11 protein (Fig. 3C) and a defined DNA substrate that mimicked reversed forks (Fig. 3D). Electrophoretic mobility shift assay (EMSA) revealed that the S649A mutant markedly increased MRE11 binding affinity to DNA compared to WT, whereas S649D showed a reduced DNA binding ability compared to WT (Fig. 3E). Thus, the S649 phosphorylation controls MRE11 binding to DNA, with loss of S649 phosphorylation enhancing MRE11 DNA binding ability.

Next, we examined the effect of S649 phosphorylation on degrading DNA *in vitro*. Using *in vitro* reconstituted DNA degradation assay as described in our previous work ^56^, we found that the S649A variant showed enhanced DNA degradation activity compared to the WT, whereas the S649D variant exhibited reduced DNA degradation activity, in line with their DNA binding ability (Fig. 3F). Together with the SIRF data, these findings suggest that S649 phosphorylation primarily regulates MRE11 binding to stalled forks, thereby ensuring controlled, transient resection while preventing pathological degradation of nascent DNA.

### S649 phosphorylation is induced by elevated intracellular Ca^2+^ level and controlled by the CaMKK2-AMPKα pathway to promote fork stability

**Figure 4:**
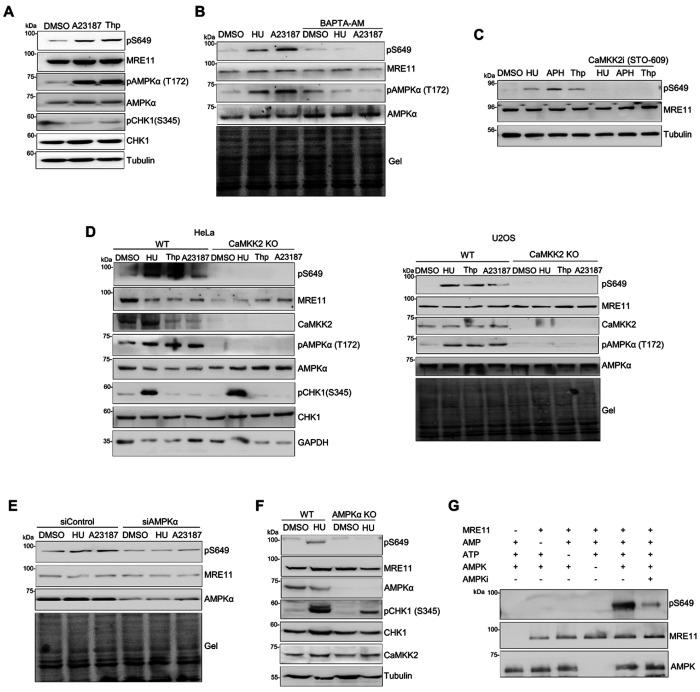
MRE11 S649 phosphorylation responds to elevated intracellular calcium levels and is controlled by the Ca^2+^-CaMKK2-AMPKα pathway. Western blot analysis of whole-cell lysates under the following experimental conditions are shown. **A.** U2OS cells treated with the calcium ionophore A23187 (1 μM, 1 h) or thapsigargin (1 μM, 1 h). **B.** U2OS cells treated with HU (4 mM, 3 h) or A23187 (1 μM, 1 h) with or without BAPTA-AM (50 μM). Total protein loading was assessed using stain-free gel imaging prior to transfer (gel). **C.** U2OS cells treated with HU (4 mM, 3 h), aphidicolin (2 μg/mL, 3 h), or thapsigargin (1 μM, 1 h) with or without the CaMKK2 inhibitor STO-609 (50 μM). **D.** WT and CaMKK2 KO HeLa and U2OS cells following treatment with HU (4 mM, 3 h), thapsigargin (1 μM, 1 h), or A23187 (1 μM, 1 h). **E.** pS649 levels in U2OS cells transfected with non-targeting control siRNA or AMPKα siRNA and treated with HU (4 mM, 3 h) or A23187 (1 μM, 1 h). Total protein loading was assessed using stain-free gel imaging prior to transfer (gel). **F.** pS649 in WT and AMPKα KO HeLa cells treated with HU (4 mM, 3 h). **G.** In vitro kinase assay. Recombinant MRE11 protein was incubated with AMPKα kinase in the kinase reaction buffer with or without AMPKαi. MRE11 phosphorylation was detected with the anti-pS649 antibody after SDS-PAGE.

Since HU treatment increases intracellular Ca^2+^ level ^50, 51^, we next determined whether the S649 phosphorylation was regulated by intracellular Ca^2+^ levels. To increase intracellular Ca^2+^ level without HU treatment, we treated cells with calcium ionophore A23187, which facilitates the transport of extracellular Ca^2+^ across the plasma membrane ^57^, and thapsigargin, which inhibits sarco-endoplasmic reticulum Ca^2+^-ATPase (SERCA) pumps and causes release of Ca^2+^ from the endoplasmic reticulum to the cytoplasm ^58^. In agreement with previous reports, elevating intracellular Ca^2+^ levels led to the activation of the CaMKK2-AMPKα pathway, shown by the phosphorylation of T172 of AMPKα (Fig. 4A). Elevation of intracellular Ca^2+^ levels did not activate CHK1, indicating that increased intracellular Ca^2+^ is not sufficient to induce replication stress (Fig. 4A). Treatment with either A23187 or thapsigargin robustly induced S649 phosphorylation of endogenous MRE11 in the absence of replication stress (Fig. 4A). Pretreatment of cells with the cell-permeable Ca^2+^ chelator BAPTA-AM reduced HU- and A23187-induced S649 phosphorylation to basal level (Fig. 4B), suggesting that S649 phosphorylation is calcium-dependent.

We hypothesized that the calcium/calmodulin-dependent kinase CaMKK2 might participate in regulating S649 phosphorylation. To test this, we pretreated cells with the CaMKK2 inhibitor STO-609 prior to treating cells with HU, APH, or thapsigargin. As expected, HU, APH, or thapsigargin treatment induced S649 phosphorylation without STO-609. Such phosphorylation was completely abolished by STO-609 treatment (Fig. 4C). Moreover, CaMKK2 KO completely abolished S649 phosphorylation of endogenous MRE11, validating the involvement of CaMKK2 in controlling S649 phosphorylation (Fig. 4D).

As AMPKα is a well-characterized downstream kinase of CaMKK2, we next tested whether it mediated S649 phosphorylation. Knocking down AMPKα significantly reduced both HU- and Ca^2+^-ionophore induced S649 phosphorylation (Fig. 4E). Moreover, AMPKα KO abolished S649 phosphorylation (Fig. 4F). Together, these results suggest that S649 phosphorylation is controlled by the CaMKK2-AMPKα axis.

### MRE11 S649 can be directly phosphorylated by AMPKα in vitro

Next, to determine whether AMPKα can directly phosphorylate S649, we performed *in vitro* kinase assay using recombinant AMPKα kinase (AMPKα1/β1/γ2) and recombinant MRE11 protein (Fig. 4G). Purified recombinant AMPKα complex is typically inactive, and can be activated by AMP allosterically ^59, 60, 61^. Following the incubation of MRE11 protein with AMPKα, a western blot was performed to detect S649 phosphorylation using the anti-pS649 antibody. We detected a robust S649 phosphorylation after the incubation of MRE11 protein with AMPKα in the presence of AMP, whereas without AMP, no S649 phosphorylation was detected (Fig. 4G, lane 5 vs lane 2). Addition of an AMPKα inhibitor in the reaction inhibited the phosphorylation (Fig. 4G, lane 6). Together, our results suggest that MRE11 can be directly phosphorylated by AMPKα at S649.

### MRE11 S649 phosphorylation is controlled by ATR and ATM but independent of CHK1

**Figure 5:**
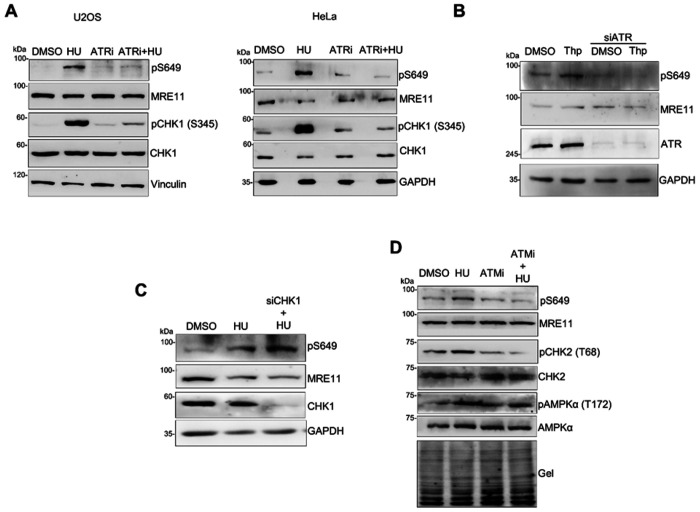
Replication stress-induced S649 phosphorylation is controlled by ATR and ATM but not CHK1. Western blot analysis of whole-cell lysates under the following experimental conditions are shown. **A.** ATR inhibition abolishes HU-induced S649 phosphorylation. pS649 levels were detected in HU-treated (4 mM, 3 h) U2OS and HeLa cells with or without ATRi VE-821 (20 μM). **B.** ATR depletion abolishes Ca^2+^-induced S649 phosphorylation. pS649 levels were detected in U2OS cells transfected with non-targeting siRNA or ATR siRNA and treated with thapsigargin (1 μM, 1 h). **C.** CHK1 depletion does not reduce S649 phosphorylation. pS649 levels were detected in U2OS cells transfected with non-targeting siRNA or CHK1 siRNA and treated with HU (4 mM, 3 h). **D.** ATM inhibition abolishes HU-induced S649 phosphorylation but does not affect AMPKα activation. pS649 and p AMPKα(T172) in U2OS cells treated with HU (4 mM, 3 h) with or without ATMi (KU55933, 1 μM). Total protein loading was assessed using stain-free gel imaging prior to transfer (gel).

To determine whether the ATR kinase regulates the phosphorylation of S649 under replication stress, we treated cells with HU along with the ATR kinase inhibitor VE-821. While ATR inhibition by VE-821 alone did not affect the basal level of S649 phosphorylation without HU treatment, VE-821 pretreatment markedly diminished HU-induced S649 phosphorylation (Fig. 5A). In addition, ATR depletion with siRNA significantly reduced thapsigargin-induced S649 phosphorylation (Fig. 5B). These results indicate that ATR kinase activity is required for S649 phosphorylation in response to replication stress.

Given that CHK1 is a downstream substrate of ATR, we next investigated whether CHK1 regulated S649 phosphorylation. Surprisingly, CHK1 depletion did not alter HU-induced S649 phosphorylation, indicating that S649 phosphorylation is independent of CHK1 (Fig. 5C).

It has been reported that ATM phosphorylates MRE11 at S676/S678 under replication stress, priming subsequent phosphorylation of multiple SQ/TQ sites on MRE11 that is necessary for promoting dissociation of MRE11 from stalled replication forks ^47^. We therefore tested whether ATM was required for S649 phosphorylation. Cells were pretreated with the ATM kinase inhibitor KU55933, followed by HU treatment. We observed that KU55933 significantly reduced HU-induced S649 phosphorylation, indicating that ATM activity is necessary for S649 phosphorylation (Fig. 5D). We also observed that while ATMi abolished CHK2 activation as expected, it had no effect on HU-induced AMPKα activation, as shown by unchanged pAMPKα (T172) in ATMi treated cells (Fig. 5D), implying that ATM is not involved in activating the calcium-responsive CaMKK2-AMPKα pathway.

### S649 phosphorylation is downstream of S676/S678 phosphorylation of MRE11 but is required for SQ/TQ phosphorylation

**Figure 6:**
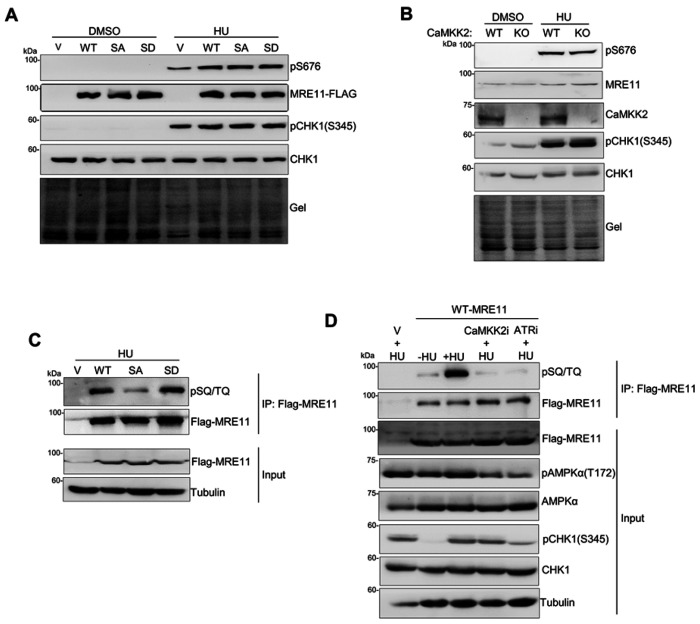
Phosphorylation of S649 stimulates the ATR-mediated SQ/TQ phosphorylation but acts downstream of ATM-mediated S676/S678 phosphorylation. **A.** pS676 western blot analysis of whole-cell lysates from HeLa cells stably expressing FLAG-WT, S649A, or S649D with and without HU treatment (4 mM, 3 hr). Total protein loading was assessed using stain-free gel imaging prior to transfer (gel). **B.** pS676 western blot analysis of whole-cell lysates from WT and CaMKK2 KO HeLa cells with or without HU treatment. Total protein loading was assessed using stain-free gel imaging prior to transfer (gel). **C.** U2OS stably expressing FLAG-tagged WT MRE11, S649A, or S649D MRE11 mutants were treated with HU (4 mM, 3 h). FLAG-MRE11 was immunoprecipitated from whole cell lysates with anti-FLAG antibody, and precipitates were used for western blotting as described in “Materials and Methods”. Phosphorylation of SQ/TQ sites was detected using the p-SQ/TQ antibody. **D.** U2OS stably expressing FLAG-WT, S649A, or S649D were treated with HU (4 mM, 3 h) with or without STO-609 (50 μM) or VE-821 (20 μM). FLAG-MRE11 was then enriched by immunoprecipitation from whole cell lysates and detected by p-SQ/TQ western blotting.

Given that MRE11 is phosphorylated at multiple sites in response to stalled replication and that these phosphorylation events are tightly regulated to ensure proper resection of stalled forks ^62^, we next investigated how these MRE11 phosphorylation events coordinately regulate fork resection. Since it has been shown that ATM-directed S676/S678 phosphorylation primes subsequent SQ/TQ site phosphorylation ^47^, we asked whether S649 phosphorylation affected S676/S678 phosphorylation. We treated cells stably expressing FLAG-tagged WT, S649A, and S649D mutants with HU, and found that S676 phosphorylation was comparable across all of them (Fig. 6A). In addition, pS676 levels were not altered in CaMKK2 KO cells relative to WT cells (Fig. 6B). Together with the result that ATMi treatment reduced S649 phosphorylation (Fig. 5D), these results indicate that ATM-mediated S676/S678 phosphorylation likely occurs upstream of CaMKK2/AMPKα-mediated S649 phosphorylation.

Next, we examined whether S649 phosphorylation affected ATR-mediated phosphorylation at SQ/TQ sites. The p-SQ/TQ antibody used in this study could not detect robust SQ/TQ phosphorylation signals from whole-cell lysates. We therefore enriched MRE11 with immunoprecipitation from cells stably expressing FLAG-tagged MRE11 using anti-FLAG antibody, and used enriched MRE11 from precipitates in western blotting analysis. As shown in Fig. 6C, WT-MRE11 or S649D displayed robust SQ/TQ phosphorylation, whereas the S649A mutant showed markedly reduced SQ/TQ phosphorylation (Fig. 6C). In addition, inhibition of CaMKK2 with STO-609, which suppresses S649 phosphorylation, significantly diminished SQ/TQ phosphorylation (Fig. 6D). Together, these findings indicate that phosphorylation at S649 is required for efficient phosphorylation at SQ/TQ sites.

### CaMKK2-AMPKα activation by replication stress is dependent on ATR

Since three different kinase signaling pathways involving ATR, ATM, and CaMKK2-AMPKα regulate MRE11 activity at stalled forks, we investigated the relationship between these three pathways. As shown in Figs. 4D and 4F, HU-induced CHK1 phosphorylation was largely unaffected in CaMKK2 KO cells (Fig. 4D), and only minimally diminished by AMPKα KO (Fig. 4F). These results indicate that the CaMKK2-AMPKα axis unlikely regulates the canonical ATR-CHK1 signaling under replication stress.

**Figure 7:**
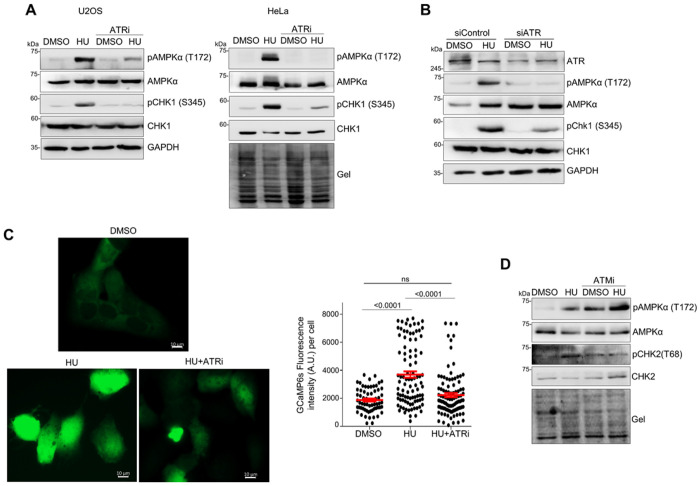
ATR regulates the CaMKK2-AMPKα pathway by regulating intracellular calcium dynamics. Western blot analysis of whole-cell lysates under the following experimental conditions are shown. **A.** pAMPKa(T172) levels in U2OS and HeLa cells treated with HU (4 mM, 3 h) with or without ATRi VE-821 (20 μM). **B.** pAMPKa(T172) in HU-treated U2OS cells (4 mM, 3 h) after ATR depletion. **C.** GCaMP6s reporter assay showing the elevation of intracellular Ca^2+^ in HU-treated U2OS cells with or without VE-821 (20 μM). Images were taken 15 min after HU treatment. GCaMP6 fluorescent signals in each cell were quantified and plotted. Scale bar: 10 μm. **D.** pAMPKa(T172) in U2OS cells treated with HU (4 mM, 3 h) with or without ATMi KU55933 (1 μM).

In contrast, when ATR kinase activity was inhibited, levels of HU-induced AMPKα phosphorylation (pT172) dropped significantly, and this reduction was also observed after ATR depletion (Figs. 7A, 7B), indicating that replication stress-induced AMPKα activation depends on ATR function. Since AMPKα is activated in response to elevated intracellular Ca^2+^, we examined whether ATR might influence intracellular Ca^2+^ concentration. To test this, we used cells stably expressing the GFP-based calcium reporter GCaMP6s, in which the GFP intensity correlates with intracellular Ca^2+^ levels ^63^. Live-cell imaging revealed that HU quickly triggered a noticeable rise in intracellular Ca^2+^ (Fig. 7C). However, ATR inhibition abolished this calcium increase (Fig. 7C). In contrast, ATM inhibition did not alter the HU-induced AMPKα phosphorylation, indicating that ATM does not directly control the CaMKK2-AMPKα pathway (Figs. 7D, 5D). These results indicate that ATM and CaMKK2-AMPKα axis are likely two independent replication stress response signaling pathways.

### Loss of S649 phosphorylation confers PARPi sensitivity

**Figure 8:**
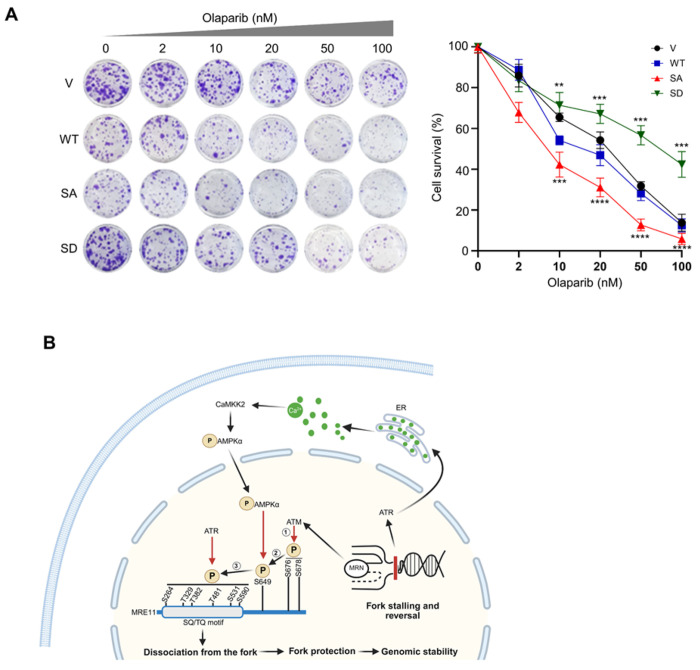
MRE11 S649 phosphorylation regulates PARPi sensitivity. **A.** Colony formation assay of U2OS cells stably expressing WT-MRE11, S649A or S649D mutants with olaparib treatment. Data represent means ± SEM from three independent experiments. **B.** Proposed model for the coordinated regulation of MRE11 at stalled replication forks. Upon fork stalling, MRN binds to the se-DSB formed at the reversed fork, activating ATM, which leads to S676/S678 phosphorylation. Simultaneously, replication stress induces ATR activation and increases intracellular Ca^2+^, activating the CaMKK2-AMPKα pathway. The S676/S678 phosphorylation primes CaMKK2-AMPKα-dependent phosphorylation at S649, which then facilitates subsequent ATR-dependent phosphorylation at SQ/TQ motifs on MRE11. These phosphorylation events restrict MRE11 binding at stalled forks, thereby preventing excessive NSD and maintaining fork stability.

To investigate whether S649 phosphorylation plays a role in cellular sensitivity to PARPi, cells expressing vector control, WT-MRE11, S649A, or S649D were treated with increasing concentrations of olaparib and subjected to colony formation assay. Vector control and WT-MRE11 cells exhibited moderate dose-dependent reductions in survival, whereas cells expressing S649A showed markedly increased sensitivity (Fig. 8A). In contrast, cells expressing S649D displayed significantly better survival (Fig. 8A). These results indicate that S649 phosphorylation protects cells from PARP inhibition.

## Discussion

Protecting stalled replication forks from uncontrolled nucleolytic degradation is critical for preserving genomic stability. Replication stress initiates a coordinated protective response involving both ATR and ATM kinases to safeguard stalled forks ^43, 64, 65, 66, 67, 68, 69^. In addition to these canonical damage response pathways, recent studies have identified a Ca^2+^-responsive CaMKK2-AMPKα signaling pathway that protects replication forks ^50, 51^. The MRN complex plays important roles in maintaining genome stability under replication stress. In particular, the nuclease activity of MRE11 needs to be tightly regulated to ensure limited end processing at stalled forks in order to prevent NSD ^34, 35^. Previous study reports that unphosphorylated MRN is recruited to se-DSBs formed at regressed arms of stalled forks, where it activates ATM and ATR, leading to ATM-dependent phosphorylation of MRE11 at S676/S678 and ATR-dependent phosphorylation at SQ/TQ motifs. Such modification regulates MRE11 activity at stalled forks, ensuring proper resection that facilitates replication restart while maintaining fork stability ^47^. In this study, we identify that the S649 residue in the DNA-binding domain of MRE11 is phosphorylated in response to replication stress. Our data support a stepwise model in which ATR and ATM are functionally linked to the CaMKK2-AMPKα pathway to control MRE11 binding to stalled forks. In this model, unphosphorylated MRE11 is initially recruited to se-DSBs at reversed forks to initiate limited resection and activate ATM, which phosphorylates S676/S678 sites. Fork stalling also activates ATR-CHK1, and a parallel intracellular Ca^2+^ signaling in which HU elevates intracellular Ca^2+^ via Ca^2+^ release from ER, leading to the activation of the CaMKK2-AMPKα pathway. The S676/S678 phosphorylation primes the phosphorylation of S649 through the Ca^2+^-CaMKK2-AMPKα axis, which reduces MRE11 binding at stalled forks and facilitates subsequent ATR-dependent phosphorylation of the SQ/TQ motif, promoting timely dissociation of MRE11 and restricting excessive nucleolytic processing of stalled forks, thereby maintaining fork integrity (Fig. 8B). Consistently with this stepwise model, the phospho-inactive S649A mutant displays enhanced DNA binding and degradation abilities, whereas the phospho-mimetic S649D variant shows reduced DNA binding and degradation (Figs. 2 and 3). Moreover, ATM activity is necessary for HU-induced S649 phosphorylation (Fig. 5D). In contrast, S649 phosphorylation has no effect on the established ATM-dependent priming phosphorylation at S676/S678 (Fig. 6A), while it is required for efficient phosphorylation of the SQ/TQ motif mediated by ATR (Figs. 6C, 6D). Together, these findings define S649 phosphorylation as a critical regulatory node that integrates canonical DNA damage signaling with calcium-dependent pathways to protect replication fork stability.

Our findings position the CaMKK2-AMPKα pathway as a central signaling hub that integrates replication stress signaling with calcium signaling to coordinately regulate multiple fork-associated nucleases and protection factors. Previous studies have shown that the CaMKK2-AMPKα pathway protects stalled replication forks by regulating EXO1 and CST ^50, 51^. Our study uncovers MRE11 as the third replication fork effector controlled by this calcium-responsive pathway (Figs. 2 and 3). Notably, these three substrates converge on a shared biological function, that is, restraining aberrant nucleolytic processing of stalled forks, suggesting that the Ca^2+^-sensing CaMKK2-AMPKα axis likely acts as a broader regulatory module that coordinates multiple nucleases and fork protection factors to preserve replication fork integrity. Further studies are needed to test this notion and identify additional fork proteins regulated by this pathway in response to replication stress.

Notably, although EXO1, STN1, and MRE11 are all phosphorylated by the CaMKK2-AMPKα pathway in response to calcium elevation, the mechanisms regulating their phosphorylation are distinct. EXO1 is directly phosphorylated at S746 by AMPKα, which inhibits EXO1 recruitment to stalled forks. ATR-CHK1 contributes in parallel to phosphorylation of the same site ^50^. In contrast, CaMKK2 directly phosphorylates STN1 at S96 within its intrinsically disordered region, and this modification occurs independently of AMPKα. This modification promotes STN1 localization to stalled forks to suppress NSD ^51^. Similar to EXO1, STN1 can also be phosphorylated by ATR-CHK1 in parallel at the same residue ^51^. Interestingly, this study finds that the MRE11 S649 phosphorylation depends on CaMKK2 and AMPKα, but is independent of CHK1. Together, these findings indicate that the downstream signaling of the CaMKK2 pathway diverges depending on the target protein and can engage distinct downstream kinases to modulate activities of different fork protection factors. Moreover, the relationship between ATR/CHK1 and CaMKK2-AMPKα pathways also varies among targets. While EXO1 and STN1 can be phosphorylated by these two pathways in parallel, CHK1 is dispensable for MRE11 phosphorylation (Fig. 5C). These findings highlight a versatile and complex signaling network that regulates nucleases and fork protectors during replication stress.

Our data reveal an unexpected connection between ATR, but not ATM, and the CaMKK2-AMPKα pathway. Although ATR is established as the central regulator of replication stress response, its potential role in modulating intracellular Ca^2+^ signaling during replication stress remains unclear. Replication fork stalling is generally viewed as a nuclear event governed by checkpoint kinases, whereas calcium signaling is often associated with cytoplasmic pathways. Existing models of replication fork protection treat ATR checkpoint signaling and the Ca^2+^-responsive CaMKK2-AMPKα signaling as largely independent pathways. Consistent with this view, previous work reported that ATR does not regulate CaMKK2-AMPKα signaling ^50^. However, our data consistently support a requirement for ATR in activating this pathway under the conditions examined in our study. ATR inhibition eliminates replication stress-induced intracellular Ca^2+^ rise (Fig. 7C) and attenuates AMPKα activation (Figs. 7A, 7B). It is possible that differences in experimental systems or cellular background may influence ATR activation of CaMKK2-AMPKα. In contrast, CaMKK2 KO or inhibition has no effect on ATR-CHK1 signaling axis (Figs. 4D, 6B, 6D), and elevating intracellular Ca^2+^ alone is insufficient to activate ATR in the absence of replication stress (Figs. 4A, 4D). Together, these findings suggest that ATR likely functions as an upstream licensing factor for the Ca^2+^-CaMKK2-AMPKα pathway at stalled forks. Following this initial step, ATR-CHK1 and CaMKK2-AMPKα operate in parallel to stabilize forks and regulate fork-associated proteins. These observations imply that ATR perhaps sits high in the replication stress response hierarchy, coordinating multiple downstream stress‑adaptation pathways, including CHK1, replication origin control, and intracellular Ca^2+^ dynamics (Fig. 8B).

In conclusion, our study uncovers a phosphorylation-based regulatory circuit that integrates ATM, ATR, and calcium signaling to fine-tune MRE11 activity at stalled replication forks. S649 serves as a key switch that limits MRE11 association with stalled forks and prevents excessive fork degradation, thereby preserving genome stability and influencing cellular responses to PARP inhibition. Future studies will be needed to elucidate the structural basis by which S649 modification alters MRE11-DNA interaction. Given the impact of S649 status on PARPi sensitivity (Fig. 8A), targeting components of the CaMKK2-AMPKα-MRE11 axis may offer new therapeutic strategies to modulate fork stability in BRCA-deficient and fork protection-defective cancers. In addition, these findings expand current models of replication fork protection by revealing how checkpoint signaling is integrated with calcium-dependent signaling pathways to directly control nuclease engagement at stalled forks.

## Materials and Methods

### Cell lines and cell culture

All cells were maintained in DMEM (GE Healthcare) supplemented with 10% fetal bovine serum (Atlanta Biologicals) at 37°C in a humidified 5% CO_2_ atmosphere. HeLa and U2OS cell lines were obtained from ATCC. AMPKα KO clones were described previously ^50^. CaMKK2 KO HeLa cells were constructed using lentiviral transduction carrying sgRNA sequences. Briefly, lentivirus produced from 293T cells after transfection with pSPAX2, pCMV-VSVG, and pLenti-CRISPR-v2-sgRNA targeting CaMKK2 (targeting sequence: CTCCTATGGTGTCGTCAAGT) were used to infect HeLa cells. Following puromycin selection, pooled cells were used for selection of single clones through serial dilution in 96-well plates. CaMKK2 protein expression from single cell clones was assayed by Western blotting. Genomic DNAs from single clones were also extracted and analyzed for insertion or deletion in the CaMKK2 exon/intron through PCR amplification and sequencing. Single CaMKK2 KO clones were expanded and used for this study.

### Plasmids

RNAi-resistant 2X-FLAG-tagged human WT-MRE11, S649A and S649D was cloned into pLV-puro retroviral vector using commercial gene synthesis service. For MRE11 protein purification, human MRE11 coding sequence was amplified by PCR and cloned into the pcDNA3.4 vector to generate an expression construct containing an N-terminal Flag tag and a C-terminal His_6_ tag ^56^. The S649A and S649D mutations were introduced by site-directed mutagenesis. All constructs were confirmed by Sanger sequencing to ensure that no additional changes were present.

### Transient transfection and RNAi

Transient plasmid transfections were performed using Lipofectamine^™^ 3000 reagent (ThermoFisher Scientific, #L3000008) following the manufacturer’s protocol, or using polyethylenimine (PEI). For PEI-mediated transfection, plasmid DNA and 40 μM PEI (Polysciences, #23966) were diluted separately and combined at a DNA:PEI ratio of 1:5 (w:v). The mixture was vortexed briefly and incubated for 15 minutes at room temperature. Then DNA:PEI complexes were added to cells in a dropwise manner. Cells were maintained at 37°C with 5% CO_2_, and downstream analyses were conducted 24–48 hours post-transfection.

Transient gene silencing was achieved using small interfering RNAs (siRNAs) targeting BRCA2 (5′-CAGGACACAATTACAACTAAA-3), human MRE11 (5′-ACAGGAGAAGAGATCAACT-3′), ATR (5′-GAGTTCTCAGAAGTCAACC-3′), CHK1 (5′-TTTGGTAAAGAATCGTGTC-3′), and AMPKα1 (5′-GGATCCATCATATAGTTCA-3′). A non-targeting control siRNA (sequence: 5′-AATTCTCCGAACGTGTCACGT-3′) was used as a negative control. Cells were transfected with 20 nM siRNA using the Xtreme RNAi transfection reagent (Sigma-Aldrich, #4476093001) according to the manufacturer’s protocol. Forty-eight hours after transfection, cells were harvested for Western blot analysis.

### Small molecules

The following small-molecule reagents were used in this study: hydroxyurea (Cayman Chemical, Cat# 23725); aphidicolin (Sigma-Aldrich, Cat# 38966-21-1); calcium ionophore A23187 (Sigma-Aldrich, Cat# C4403); Thapsigargin (Sigma-Aldrich, Cat# T9033); STO-609 (ApexBio, Cat# B6787); VE-821 (Sigma-Aldrich, Cat# SML1415); BAPTA-AM (Cayman Chemical, Cat# 15551); 5-ethynyl-2′-deoxyuridine (EdU, Lumiprobe, Cat# 10540); KU55933 (Sigma-Aldrich, Cat# SML1109).

### Inhibitor treatments

Cells were pretreated with the indicated inhibitors (STO-609, ATRi, ATMi, or BAPTA-AM) for 30 min prior to induction of replication fork stalling or elevation of intracellular calcium. The inhibitors were maintained in the culture medium during subsequent treatment with HU, A23187, or thapsigargin.

### Western blotting and antibodies

Protein samples were prepared and analyzed by Western blot as described previously ^70^. Briefly, cells were lysed in a buffer containing 1% CHAPS, and proteins were resolved by SDS-polyacrylamide gel electrophoresis (SDS-PAGE). Gels were transferred onto PVDF membranes, which were then blocked with 5% bovine serum albumin (BSA) or 5% non-fat milk in TBST (Tris-buffered saline, 0.1% Tween-20) for 1 hour at room temperature. Membranes were incubated with primary antibodies at 4°C overnight, washed in TBST, and then probed with horseradish peroxidase (HRP)-conjugated secondary antibodies. Signals were detected using SuperSignal^™^ West Femto (Thermo Fisher Scientific, #34095). For stain-free SDS-PAGE, 0.5% 2,2,2 Trichloroethanol (MilliporeSigma, T54801) was included in the gel during gel casting. Gels were exposed to UV light for 5 minutes prior to being transferred to PVDF membranes. After transfer, membranes were imaged with UV to reveal total proteins on membranes before blocking and incubation with antibodies. For λ-phosphatase treatment, cell lysates were treated with λ-phosphatase (Santa Cruz Biotechnology, #sc-200312A) at 30°C for 30 minutes prior to gel loading.

Primary antibodies used were: pS649 (custom made, 1:1000), MRE11 (1:1000; GeneTex, #GTX70212), FLAG (1:5000; Sigma-Aldrich, #F1804), pCHK1 (S345) (1:1000; Cell Signaling Technology, #2348), CHK1 (1:2000; Santa Cruz Biotechnology, #sc-8408), BRCA2 (1:5000; Sigma-Aldrich, # OP95), ATR (1:1000; Cell Signaling Technology, #2790), phospho-AMPKα (pThr172) (1:1000; Cell Signaling Technology, #2535), AMPKα (1:1000; Cell Signaling Technology, #2532), CaMKK2 (1:300; Santa Cruz Biotechnology, #sc-271674), phospho-RPA32 (S4/S8) (1:1000; Bethyl, A300-245A-T), RPA32 (1:1000; Bethyl, #A300-244A), phospho-CHK2 (Thr68) (1:500; Cell Signaling Technology, #2661), CHK2 (1:500; Cell Signaling Technology, #2662), phospho-(Ser/Thr) ATM/ATR substrate (1:1000; Cell Signaling Technology, #2851), phospho-MRE11 (S676) (1:1000; Cell Signaling Technology, #4859), GAPDH-HRP conjugated (1:20000; Proteintech, HRP-60004), Tubulin (1:5000; Sigma-Aldrich, #T5168), Vinculin (1:1000; Proteintech, #66305-1-Ig). Secondary antibodies: HRP-conjugated goat anti-rabbit IgG (1:15000; Vector Laboratories, #PI-1000), HRP-conjugated goat anti-mouse IgG (1:10000; Vector Laboratories, #PI-2000).

### DNA fiber assay

DNA fiber assay was performed according to the published protocol ^71^. Briefly, cells in exponential growth were sequentially pulse-labeled with two thymidine analogs: first with 50 μM chlorodeoxyuridine (CldU) for 20 minutes, then with 250 μM iododeoxyuridine (IdU) for 20 minutes. Immediately after labeling, cells were washed with PBS and then exposed to 4 mM HU for 3 hours to induce replication fork stalling. In experiments for testing fork protection, endogenous BRCA2 and MRE11 were depleted using respective siRNA. Forty-eight hours after siRNA transfection, cells were treated with HU (4 mM, 3 h). Labeled cells were harvested and resuspended in 12 μL of lysis buffer (200 mM Tris-HCl pH 7.5, 50 mM EDTA, 0.5% SDS). The cell suspension was applied to a glass microscope slide and incubated in a humid chamber for 2 minutes to lyse cells. The slide was then gently tilted at approximately 15° to allow genomic DNA to spread down the slide by gravity. The DNA fibers were air-dried for ~ 30 minutes, after which the slide was fixed in a 3:1 mixture of methanol:acetic acid for 10 minutes. Next, the slide was immersed in 2.5 M HCl for 100 minutes to denature the DNA, followed by three rinses in PBS. The slide was then blocked with 5% bovine serum albumin (BSA) in PBS for 30 minutes. DNA tracks were immunostained by incubating the slide for 1 hour at 37°C in a humid chamber with primary antibodies against CldU (1:500, Abcam #ab6326) and IdU (1:50, BD Biosciences #347580). After three PBS washes, the appropriate secondary antibodies (1:500, Alexa-488 anti-rat IgG, Thermo Fisher #A11006, and 1:500, Alexa-568 anti-mouse IgG, Thermo Fisher #A11031) were applied for 1 hour at 37°C. The slides were then washed with PBS, air-dried in the dark, and mounted with a coverslip using a DAPI-free mounting medium (Vector Laboratories, H-1000). Images of DNA fibers were captured with Zeiss Axio Observer 7 fluorescence microscope using a 40× objective. Fiber tract lengths were measured using the ZEN software 10.1, and approximately 200 DNA fibers were analyzed per condition. Data were plotted using GraphPad Prism 10. For critical experiments, two researchers analyzed the fiber images to avoid bias. All DNA fiber assays were performed at least three independent experiments to ensure reproducibility.

### SIRF assay

SIRF assay was performed as described previously ^55, 56^. Exponentially growing cells were seeded on chamber slides and labeled with 125 μM EdU for 8 minutes. After a PBS wash, cells were treated with 4 mM HU for 3 hours. Cells were then pre-permeabilized with 0.25% Triton X-100 for 2 minutes before being fixed with 2% paraformaldehyde for 15 minutes at room temperature, followed by three times wash with PBS. Next, the cells were permeabilized again with 0.25% Triton X-100 for 15 minutes. Incorporated EdU was detected by a click chemistry reaction: slides were incubated for 1 hour at 37°C in a humid chamber with 2 mM CuSO_4_, 10 μM biotin-azide, and 100 mM sodium ascorbate. After the click labeling, slides were washed three times in PBS. Cells were then blocked with blocking buffer (10% BSA, 0.1% Triton X-100 in PBS) for 1 hour at 37°C. Primary antibodies anti-FLAG (1:200; Sigma-Aldrich, #F1804) and anti-biotin (1:200; Cell Signaling Technology, #5597) were diluted in Duolink antibody diluent (Sigma-Aldrich, #DU092101) and incubated with cells at 4°C overnight. On the next day, PLA was performed using the Duolink In Situ Red Starter Kit (Sigma-Aldrich, #DU092101) according to the manufacturer’s instructions. Slides were air-dried and mounted with antifade medium containing DAPI (Vector Laboratories, #H-1200-10). Fluorescence images were acquired on a Zeiss Axio Observer 7 fluorescence microscope at 63X magnification and analyzed with ZEN software 10.1; quantitative results were plotted using GraphPad Prism 10. For quality control, two researchers independently analyzed the PLA signals. All SIRF assays were repeated in three independent experiments.

### Immunoprecipitation (IP)

IP assays were used to enrich FLAG-MRE11 from whole cell lysates to monitor the phosphorylation of SQ/TQ sites on MRE11. U2OS cells stably expressing vector, WT-MRE11, S649A or S649D were seeded in 60 mm dish. Next day, cells were treated with 4 mM HU for 3 hours to induce replication stress. Calyculin A (5 nM, Sigma Aldrich, #C5552) was added 15 minutes prior to cell collection. Collected cells were lysed in ice-cold lysis buffer (1% NP-40, 0.1% SDS, 50 mM Tris-HCl pH 7.6, 150 mM NaCl, 1 mM EDTA) supplemented with protease inhibitors (Sigma-Aldrich, #P2714). Lysates were sonicated on ice (3 × 5-second pulses with 1-minute intervals) and centrifuged at ~ 17,000 × g for 10 minutes at 4°C. Supernatants were incubated with anti-FLAG agarose affinity resin (Millipore Sigma, #A2220) overnight at 4°C on rocker. Next day, sample tubes were centrifuged at 900× g for 3 minutes at 4°C and supernatant was discarded. Beads were washed two times with ice-cold lysis buffer. Bound proteins were eluted by boiling the beads in SDS sample buffer for 10 minutes at 68°C. The samples were analyzed by SDS-PAGE followed by Western blotting. All IP experiments were performed at least three times to ensure reproducibility.

### Immunofluorescence (IF) staining

U2OS cells were grown on chamber slides and treated with 4 mM HU for 3 hours. Cells were then fixed with 4% paraformaldehyde for 15 minutes at room temperature and subsequently permeabilized with 0.15% Triton X-100 for 15 minutes. After three washes in PBS (5 minutes each), cells were blocked in 10% BSA (in PBS) for 1 hour at 37°C in a humidified chamber. γ-H2AX (pSer139) (1:200; Cell signaling, #2577) was applied and incubated overnight at 4°C. The slides were then washed three times in PBS (5 minutes each) and incubated with Dylight 649-conjugated anti-rabbit secondary antibody (1:500; Thermofisher Scientific, #35565) for 1 hour at room temperature. After three more 5-minute washes in PBS, the slides were mounted with a DAPI-containing anti-fade medium (Vector Laboratories, #H-1200-10). Fluorescence images were captured Zeiss Axio Observer 7 fluorescence microscope and analyzed with ZEN software 10.1.

### Live cells imaging of Ca^2+^ reporter

U2OS cells stably expressing GCaMP6s were treated with HU (4 mM) in presence or absence of VE-821 (20 μM). Images were acquired at every 15 min interval for 1 h using Zeiss Axio Observer 7 fluorescence microscope and processed with ZEN software 10.1.

### Colony formation assay

Cells were plated at a low density of 400 cells per well in 6-well plates and allowed to attach. The following day, cells were exposed to various concentrations of Olaparib. After Olaparib treatment, cells were then incubated for approximately 15 days to allow colonies to form from surviving cells. Colonies were fixed in a 1:7 mixture of acetic acid:methanol and stained with 0.5% crystal violet solution. The number and size of colonies were recorded.

### Expression and purification of human MRE11 recombinant protein

Recombinant human MRE11 protein (WT, S649A and S649D) was expressed in human Expi293F cells (ThermoFisher) and purified following previously established procedures ^56^. Briefly, MRE11 protein in harvested cell lysates was captured by Ni-NTA (QIAGEN, #30230) and anti-Flag M2 (Sigma-Aldrich, #A2220) affinity chromatography. The affinity-purified fractions were further resolved by anion-exchange chromatography on a Mono Q column (GE Healthcare, #17516601). Peak fractions containing MRE11 were pooled, concentrated, aliquoted, and stored at − 80°C.

### DNA substrate

The 5′-overhang substrate used for the EMSA and MRE11 degradation assay was generated by annealing fluorescently-labeled oligonucleotide 1 (Genomics, 5’-Cy3-GGGTGAACCTGCAGGTGGG CAAAGA) with the complementary 5′/3′-phosphorothioate-modified oligonucleotide 2 (IDT, 5’-A*C*G*C*T*GCCGAATTCTACCAGTGCCTTGCTAGGACATCTTTGCCCACCTGCAGGTT*C*A*C*C*C, * indicates phosphorothioate bond modification between two nucleotides) at a molar ratio of 1:1.2 in annealing buffer (50 mM Tris-HCl pH 7.5, 10 mM MgCl_2_, 100 mM NaCl, and 1 mM DTT). The phosphorothioate modifications at both termini were used to prevent nonspecific nuclease digestion of the substrate. Annealing reactions were carried out by heating the mixture to 80°C for 3 min, followed by incubation at 65°C for 30 min and slow cooling to room temperature. The annealed DNA substrate was subsequently purified from TBE-PAGE gels by electroelution, concentrated, and exchanged into TE buffer (10 mM Tris-HCl pH 8.0 and 0.5 mM EDTA) at 4°C using Amicon ultra-4 centrifugal filters (Millipore, NMWL 10 kDa, #UFC801024).

### Electrophoretic mobility shift assays

The above DNA substrate (5 nM) was incubated with the indicated amounts of WT-MRE11, S649A, or S649D proteins in 10 μl of buffer A (35 mM Tris-HCl pH 7.5, 1 mM DTT, 50 mM KCl, 0.1 μg/μl BSA, 4 mM MgCl_2_, and 1 mM ATP) at 37°C for 30 min. Following incubation, the reaction products were resolved on an 8% TBE-PAGE in 1× TBE running buffer (89 mM Tris, 89 mM borate, and 2 mM EDTA, pH 8). Electrophoresis was performed at 80 V for 60 min at 4°C. Fluorescent signals were detected using an Amersham^™^ Typhoon^™^ Biomolecular Imager (Cytiva) with a Cy3 570BP20 560–580 nm filter. The signal intensities of DNA species were normalized to the corresponding controls and quantified using ImageQuant^™^ TL.

### MRE11 degradation assay

For time-course assays, a fluorescently labeled 5′-overhang DNA substrate (58 nM) was incubated with 50 nM WT-MRE11, S649A, or S649D proteins in buffer A (10 μL) containing 2.5 mM MgCl_2_ and 1 mM MnCl_2_ at 37°C for the indicated times. Reactions were terminated by adding 2.5 μL termination buffer (0.4% SDS, 0.24 M EDTA, and 3 mg/mL proteinase K) and incubated at 37°C for 15 min. Samples were then mixed with 2× denaturing loading dye (95% formamide, 0.1% Orange G, 10 mM Tris-HCl pH 7.5, 1 mM EDTA, and 12% Ficoll PM400) and heated at 95°C for 10 min. Reaction products were separated on a 27% denaturing TBE-urea gel (7 M urea) in 1× TBE at 300 V for 60 min at 55°C. Fluorescent signals were visualized using an Amersham^™^ Typhoon^™^ Biomolecular Imager with a Cy3 (570BP20, 560–580 nm) filter. The signal intensities of DNA species were normalized to the corresponding controls and quantified using ImageJ.

### In vitro kinase assay

Recombinant MRE11 protein was incubated with recombinant AMPK complex (α1/β1/γ2) (Active Motif, Cat#81437) in kinase reaction buffer (5 mM MOPS pH 7.2, 5 mM MgCl_2_, 1 mM EGTA, 0.4 mM EDTA, and 0.05 mM dithiothreitol) with 100 μM ATP (Tokyo Chemical Industry, Cat#A0157) and 50 μM AMP (MedChemExpress, Cat#HY-A0181) in a final reaction volume of 20 μL. The AMPKα inhibitor dorsomorphin (MedChemExpress, Cat#HY-13418A) was added to a final concentration of 10 μM. Reactions were mixed at room temperature and incubated at 30°C for 30 minutes. Reactions were terminated by adding 2× SDS sample buffer and heating at 68°C for 10 minutes. Samples were loaded on SDS-PAGE and transferred to PVDF membranes for immunoblot analysis. Phosphorylation of MRE11 at S649 was detected using a phospho-specific antibody against pS649, while total MRE11 was detected using the anti-MRE11 antibody.

### Statistics

Statistical analyses of biochemical data were conducted using GraphPad Prism 10 (GraphPad software). Data normality was evaluated by the Shapiro-Wilk test, and homogeneity of variance between groups was assessed using the Brown-Forsythe test. Statistical significance among multiple groups was determined by one-way ANOVA followed by Tukey’s post hoc test.

## Data Availability

All data needed to evaluate the conclusions in the paper are present in the paper and/or the Supplementary Materials.
